# Application of a cost calculation approach in medical oncology: case of Hassan II university hospital in Morocco

**DOI:** 10.1186/s12962-023-00462-8

**Published:** 2023-08-11

**Authors:** A. Benabdallah, T. Jellouli

**Affiliations:** Faculty of Law, Economic and Social Sciences, Sidi Mohammed Ben Abdellah University, Fez, Morocco

**Keywords:** Cost calculation, Hospital, RVU method, Medical oncology, Top-down approach

## Abstract

**Background:**

In Morocco, hospitals do not have cost accounting systems that allow them to produce reliable budget forecasts to know and justify the costs of their operations. Moroccan hospitals are thus among the public organizations that do not know the cost of their services. Faced with the lack of data on the cost of care for cancer patients, this research aims to implement an approach to calculate the cost of services provided at the level of the medical oncology department of the Hassan II University Hospital in Fez. The objective is to provide data that can be used in the pricing and financing process of the different services provided in medical oncology.

**Methods:**

In this research work, we proposed a top-down costing approach. Two main qualitative data collection tools were used: observation and document analysis. We used mainly and Excel for data processing to determine costs and related statistics.

**Results:**

The result of this study shows the possible application of a top-down approach to cost calculation which consists in determining the cost per department (oncology service) and per product (chemotherapy act, transfusion, ascites puncture, Inpatient day). Two main methods were mainly used, namely the volume-based allocation method and the method relative value units (RVU). The proposed top-down cost calculation approach has the advantage of being easy to implement, but on the other hand, it is not very accurate in producing results on the actual cost. The data from this study can be exploited to revise the prices of procedures provided in medical oncology.

## Introduction

The hospital sector has been considered excessively bureaucratic, rigid, costly, non-innovative and with an overly centralized hierarchical structure. Hospital management methods are mainly oriented towards the search for state subsidies. Similarly, the administrative and budgetary procedures in force have not encouraged the actors to take charge of their institutions.

In this respect, the management of hospitals has also been strongly criticized by the New Public Management (NPM) movement. Thus, the hospital reform undertaken during the 1980s in several high-income countries aimed to control hospital expenditure, through the deployment of accounting and financial control tools in these establishments, as well as the accountability of managers and health professionals [1]. The development of management accounting in these countries is older, particularly in the Anglo-Saxon countries, where the academic literature in this field is very rich. Information on the costs of hospital services has been used both by funders to correctly establish fee schedules and funding for health care providers, and by hospital managers to ensure the financial health of their institution by comparing the costs of health care services with the payments received [2].

Costing in healthcare is currently one of the most important tools for hospital decision-makers in high-income countries, allowing them to efficiently manage health care facilities and their structures. The main objectives of the hospital cost accounting system [3]:

- To identify the costs and revenues of the various functions involved in the production of care, whether they are clinical, medico-technical or administrative functions;
- To provide management control with forecasting information based on the results of cost accounting;
- To provide management with the information necessary to make management and organizational decisions.

The financial difficulties and limited resources allocated to the health system in low- and middle-income countries are an obstacle to the implementation of hospital financing reforms. It is true that cost accounting has become an established management tool, particularly with the introduction of activity-based financing, which promotes efficiency and hospital production.

Moreover, the implementation of a costing system requires significant human and technical resources, in particular an information system gathering complete clinical data by patient. However, the cost of acquiring and appropriating information systems is one of the obstacles to the introduction of hospital cost accounting in low- and middle-income countries.

In the absence of a hospital cost accounting system in Morocco, data from the billing system cannot be used to determine the real cost of the care provided. However, measuring the cost requires taking into consideration all the resources used to care for patients.

The question of how to calculate the cost of medical procedures is an innovative one in middle and low-income countries where there are no formal costing systems. Indeed, the information obtained from studies that focus on the costing of hospital services can partially fill the data gap related to the absence of hospital cost accounting.

In this respect, this research aims to explore an approach to calculate the costs of the services provided in the medical oncology department of the Hassan II University Hospital (CHU) of Fez.

The allocation of direct expenses does not pose a problem insofar as the CHU billing system allows direct tracking of patient consumption of drugs and ancillary products. The proposed calculation approach is limited to the presentation of the method of allocation of indirect costs.

Indeed, in the absence of a coding system (DRG), the studies provide either average costs per patient or indirect cost estimates [4].

## Methods

*Description of the place of study the Hassan II hospital center and the medical oncology service.*

### Description of the Hassan II hospital center

The Hassan II Hospital Center of Fez was created by virtue of the law 82 − 00 of August 30, 2001 modifying and completing the law n° 37–80 of January 15, 1983 relating to the hospital centers. It was put into operation on August 5, 2002 following the publication of the decree n°2 -86-74 of July 19, 2002 of application of the above-mentioned law.

According to the law N° 37.80, the university hospital center is a public establishment with legal personality and financial autonomy, subject to the supervision of the State. The Hassan II Hospital Center of Fez has the following mission:

- To provide medical care;
- To conduct medical research in the strict respect of the physical and moral integrity and dignity of the patients;
- To participate in university and post-graduate clinical medical and pharmaceutical education as well as in the training of paramedical personnel;

### Description of the medical oncology service

The medical oncology department was inaugurated in 2012. The department’s activities are mainly limited to the medical treatment of cancers through chemotherapy and the hospitalization of patients who are impaired or require special and palliative treatments. The Table 1 presents the medical oncology department:

**Table 1:** Presentation of the medical oncology department

The medical oncology department has the following staff: - A medical team composed by 14 contractual resident doctors, 3 volunteers, 1 specialist doctor and 5 professor doctors; - A nursing team composed of: a head nurse and 21 polyvalent nurses; - Administrative staff and agents of subcontracting companies. The medical oncology department has two units:	
Hospitalization unit with a capacity of 12 beds: - Hospitalization of patients with treatment complications and other oncological emergencies - Hospitalization of patients with treatment protocols extending to several continuous days	Day hospital with 25 chemotherapy chairs: -Administration of chemotherapy products. -Other medical acts: punctures, transfusions, administration of analgesic treatments and others.

We directed our research from the field to cancer care services where management is costly. The choice of the medical oncology service is justified by the following elements:

- In Morocco, few studies have focused on the implementation of a method for calculating the costs of services provided in medical oncology at a university hospital.
- At the level of the Hassan II University Hospital the chemotherapy activity is very old compared to the other activities such as radiotherapy and nuclear medicine. Therefore, the medical activity is well organized and controlled and the staff of the service is familiar with the procedures implemented;
- The adhesion of the staff of the oncology service and their agreement to use different data (statistics, activities and financial), necessary to conduct this study. Thus, we carried out a one-month internship in the medical oncology department;
- The processes of care at the level of the medical oncology service of Fez are well structured;
- Medical information and statistical data are well maintained by the department’s staff.

### Description of the costing approach used and the data collection method

In this research work, we proposed top-down costing approach uses traditional costing tools. Indeed, traditional costing methods (volume-based allocation, cost/expense ratio, relative value units) have proven to be very effective in the hospital context in several countries.

**Fig. 1:**
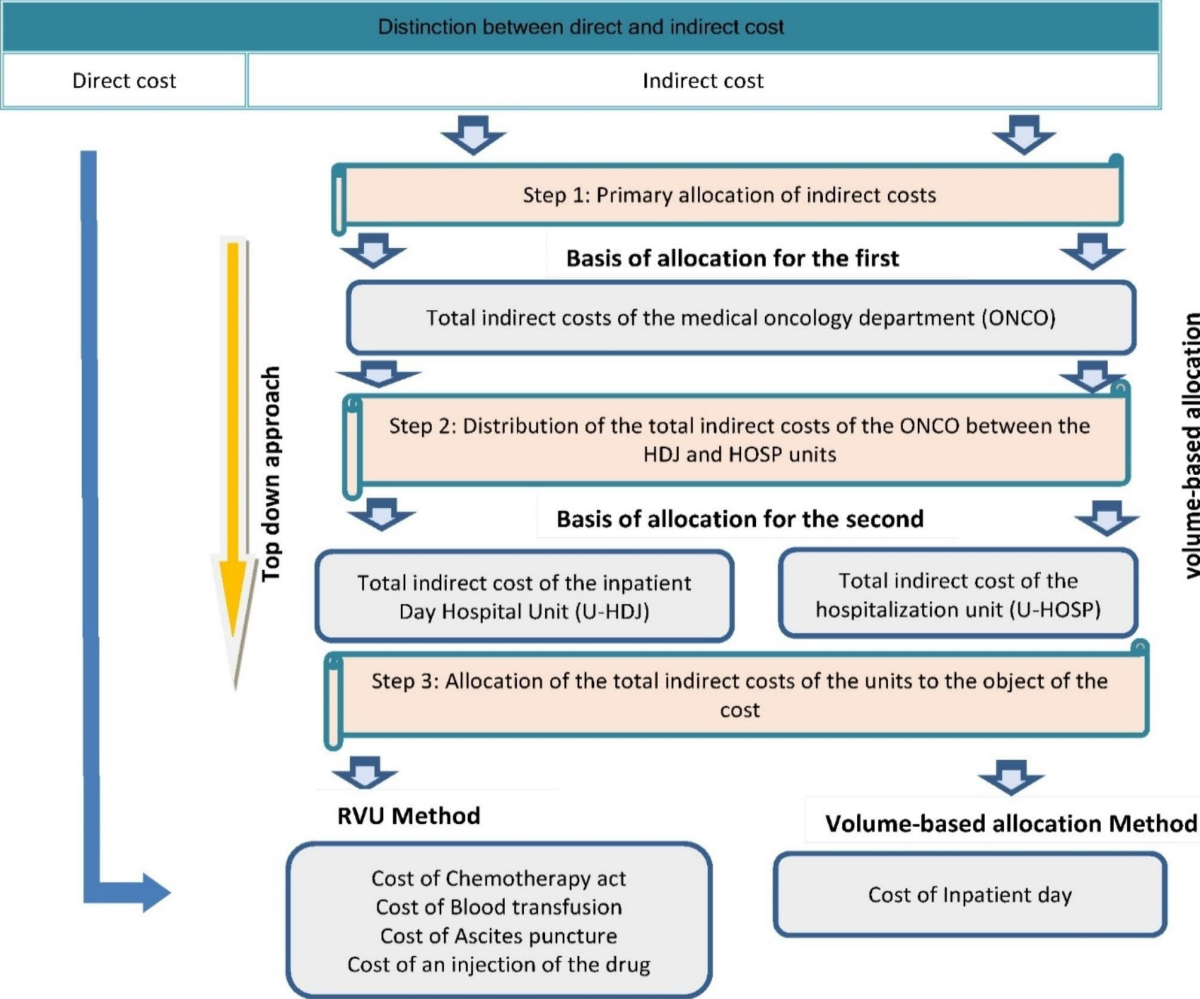
Conceptual model for calculate costs in an oncology department

The proposed calculation approach is based on three steps. The Fig. 1 presents the conceptual model for calculating the cost of chemotherapy act and other services provided in the oncology department.

In this study, direct costs were excluded because the billing system allows direct tracking of patient consumption of pharmaceutical products and medical consumables. Indeed, direct charges vary significantly from one patient to another in medical oncology.

Two main qualitative data collection tools were used: observation and document analysis. Through participant observation, we freely asked questions of the study participants in order to elicit the desired information. This observation allowed us to identify the activities, actors and resources that play a role in the care process of cancer patients in the medical oncology department of the Hassan II University Hospital in Fez.

The visit of the premises was carried out several times. We were able to see all the rooms of the day hospital and the hospitalization unit, the materials available, the staff (medical and paramedical) and the agents involved in the different activities of the oncology service.

The use of data from the different documents is often relevant in the case study. We used mainly activity data (statistics) and financial data from the oncology department. These data are mainly contained in computerized databases (Word and Excel files), but also in paper media (registers and records). The reference year used for data processing is 2021.

We used mainly Microsoft World for text entry and Excel for data processing to determine costs and related statistics.

## Results: Summary of the proposed costing approach

It was possible to apply a costing approach for medical oncology through a three-step process:

### Step 1: identification and allocation of indirect costs in the medical oncology center “ONCO”

In the first step, we identified and assigned all indirect costs to the medical oncology center (ONCO), the object of the cost analysis, using the cost drivers of this step.

We have tried to keep the current titles of the expenses as they are presented in the Hassan II University Hospital chart of accounts. The expenses of the subcontracted agents concern the cleaning of the buildings, stretchering, guarding and the outsourcing of the medical secretariat.

The Table 2 shows the basis for allocation indirect costs and the total indirect costs allocated to the ONCO.

**Table 2:** Total indirect costs allocated to the “ONCO”

Title of expenses	Basis of allocation for the first step	Amount
Subcontractor expenses	Day worked	905 174,40
Laundry and linen costs	Kg of treated linen	13 150.90
Purchase of supplies	Total value of supplies delivered	63 069.04
Food costs for patients and medical staff	Total on-call (staff) Sick Day	361 294,90
Disinfection of premises	Liter of disinfectant	17 497.23
Treatment of hospital waste	Kg of treated waste	48 172.80
depreciation of tangible capital assets	Annual allocation to departments	540 564,85
Purchase of medical gases	Number of departments	67 677.33
Postage and telecommunications	Number of departments	19 423.61
Purchases of non-stackable supplies	Number of departments	260 185,00
Personnel costs	Total annual salary	5 381 675,06
Insurance and Worker’s Compensation	Number of staff	11 404.02
Salary processing for staff	Number of staff	2 640,00
		**7 691 929,14**

### Step 2: allocation of indirect costs allocated to the oncology center (ONCO) between the Day Hospital Unit (U-HDJ) and the hospitalization unit (U-HOSP)

In a second step, we allocated the different indirect costs retained in the first step to the two units making up ONCO (U-HDJ and U-HOSP), using the allocation bases of the second step.

The Table 3 presents the allocation bases used in this second stage and the total costs allocated to the two units (HDJ and HOSP) making up ONCO:

**Table 3:** Total indirect costs per U-HDJ and U-HOSP

Nature of expenses	Basis of allocation for the second step	amount allocated per U-HDJ	amount allocated per U-HOSP
Overheads (water, electricity, telecommunications, etc.)	Total number of shifts worked	235 533,35	111 752,59
Expenses of subcontractors	Number of officers assigned	684 248,76	220 925,64
Laundry and linen costs	Number of inpatient beds	4 934,96	8 224,94
Purchase of supplies (logistics store)	Total number of shifts worked	32 671,35	30 397,69
Patient feeding and staff costs	Total number of shifts worked	74 520,00	94 872,50
Disinfection of premises	Liter of disinfectant	10 510,29	6 986,94
Treatment of hospital waste	Total number of shifts worked	32 671,35	30 397,69
Depreciation of tangible capital assets	Annual allocation (unit specified)	271 370,71	269 194,14
Total other operating expenses (a)		1 346 460,77	772 752,13
Personnel costs (salaries and allowances)	Total number of shifts worked	3 842 138,75	2 502 187,92
Insurance and Worker’s Compensation	Total number of shifts worked	7 734,34	3 669,68
Salary processing for staff	Total number of shifts worked	1 952,22	567,78
Total staff costs (b)		3 851 825,31	2 506 425,38
Total cost (a) + (b)		**5 198 286,08**	**3 279 177,51**

**Fig. 2:**
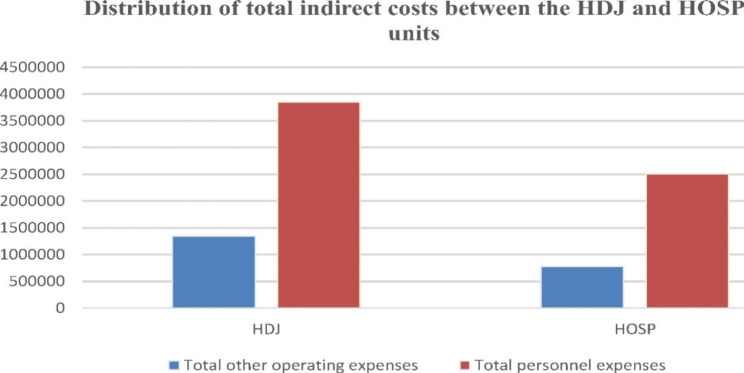
Distribution of total indirect costs between the HDJ and HOSP units

The distribution of total indirect costs allocated to the HDJ and HOSP units according to the nature of the expenses (personnel or other operating expenses) is presented in the Fig. 2:

The figure presented above shows that the costs allocated to the HDJ unit exceed those allocated to the HOSP unit. Indeed, 61.13% of the indirect costs are attributed to the HDJ unit. This percentage of costs attributed to the HDJ unit can be considered normal insofar as this unit represents the main activity of the medical oncology department.

### Step 3: calculation of the costs of medical oncology services

#### Calculation of the cost per day of hospitalization (hospitalization unit)

The cost of a day in the inpatient unit of the oncology department is calculated as the total cost allocated to this unit based on the total number of inpatient days (Table 4):

**Table 4:** Cost per day of hospitalization

Total cost allocated to the HOSP unit (a)	Number of Inpatient day (b)	cost per day (a)/(b)
3 279 177,51 DH	9125	359,36 DH

#### Calculation of the cost of medical procedures performed in the HDJ unit

In addition to chemotherapy, which is the main activity of the HDJ unit, other medical procedures are also performed: transfusion, ascites puncture and administration of drugs (mainly morphine infiltrations).

In this context, the method of relative value units is more appropriate because it allows us to differentiate and position the chemotherapy act in relation to the other medical acts performed at the HDJ level.

To do this, we first tried to measure and compare the complexity of the other medical acts in relation to the chemotherapy act by using weighting units. Thus, the total number of relative units produced by the HDJ unit is the result of this first step.

In a second step, we divided the total costs previously allocated to the HDJ unit (personnel costs and other operating costs) over the total number of relative units produced by that unit to obtain the cost per relative unit.

In a final step, the cost of the different medical procedures (excluding direct expenses: medical consumables and drugs) was obtained by multiplying the relative value allocated to each procedure by the cost of the relative unit. We will present these different steps in turn:

Referring to some works [3, 4, 5], we set up a methodology to determine the relative values of the medical procedures used at the HDJ level. The complexity of the medical procedure and the time required to perform the procedure are the variables included to assign relative weights to the different procedures.

#### Determination of time spent performing medical procedures

The time spent by a healthcare professional to perform a procedure is undoubtedly an important determinant of its value. The Table 5 presents the average time spent performing medical procedures determined from empirical observations in the HDJ.

**Table 5:** Time spent performing medical acts

Procedure	Number of observations	Average time in minutes
Chemotherapy act	20	147 mn
Blood transfusion	10	95 mn
Ascites puncture	5	103 mn
injection of the drug	10	16 mn

The table shows that the average time required to perform a chemotherapy procedure at the HDJ level exceeds that for each of the other medical procedures.

#### Measurement of the complexity of the different procedures

the time required to perform a medical act does not fully describe the professional effort required. While in some cases the time taken to perform the procedures is almost equal, the degree of intensity and effort required per unit of time varies considerably from one procedure to another. The value of a procedure will thus have to take into account the differences in complexity. This includes mainly two elements:

* The intensity of the physical and mental effort involved;

* The diagnostic skills and clinical judgments required.

To take into account the degree of complexity of the different procedures, we asked five physicians in the medical oncology department to rank on a 10-point scale the complexity of the different procedures performed in the HDJ unit. We explained to the physicians that their classification must take into account the intensity of the procedures performed, particularly the physical and mental effort involved in performing the various procedures. The Table 6 presents the average scale obtained, using the medical evaluation of the complexity of the different acts.

**Table 6:** Complexity attributed to the medical acts performed at the HDJ level

Procedure	Complexity factor (Scale from 1 to 10)
Chemotherapy act	7,5
Blood transfusion	6
Ascites puncture	5
injection of the drug	3

Analysis of the table shows that medical staff rank the complexity attributed to the chemotherapy procedure as the most important. However, the complexity per unit of time of a medical procedure thus takes into account both the time spent and the complexity factor assigned to the procedure.

#### Determining the relative value assigned to each procedure

To determine the value of each medical act, we assigned a value of 1 to the chemotherapy act, which corresponds to the main product of the HDJ. The value of the other procedures is determined by evaluating the complexity per unit of time of each of them in relation to the chemotherapy procedure. The Table 7 presents the method for determining the relative values of the other procedures.

**Table 7:** Relative value attributed to medical acts performed at the HDJ level

Procedure	Average time in mn (t)	Complexity factor (c)	Complexity per unit of time (t*c)	Relative value (with chemotherapy act as standard reference = 1.0)
Chemotherapy act	147	7,5	1102,5	1
Blood transfusion	95	6	570	0,52
Ascites puncture	103	5	515	0,47
injection of the drug	17	3	51	0,05

#### Determination of the total number of relative value units

The total number of relative value units attributed to each medical procedure is determined by multiplying the number of procedures by the relative value attributed to the procedure (Table 8):

**Table 8:** Total number of RVUs attributed to medical acts performed at the HDJ level

Procedure (act)	Number of acts (a)	Relative value attributed to the act (b)	Total number of RVUs (a)*(b)
Chemotherapy act	15 476	1	15 476,00
Blood transfusion	788	0,52	409,76
Ascites puncture	306	0,47	143,82
injection of the drug	540	0,05	27,00
			**16 056,58**

#### Calculation of cost per relative unit of value

To obtain the cost per unit of relative value of the various medical acts carried out at the HDJ level, we divided the total cost (personnel and other expenses) allocated to the HDJ on the total number of relative units produced (Table 9):

**Table 9:** Calculation of the cost per unit of relative value

Total cost (personnel and other expenses)	Total number of RVUs	cost per RVUs
5 198 286,08 DH	16 056,58	323,75 DH

#### Calculation of the cost of medical procedures performed in HDJ

Finally, the cost of the various medical acts is determined by multiplying the cost per relative unit by the relative value attributed to the act (Table 10).

**Table 10:** Calculation of the cost of medical procedures performed in HDJ

Nature of the acts	cost per RVUs	Value attributed	Cost of the acts
Chemotherapy act	323,75 DH	1	323,75 DH
Blood transfusion	323,75 DH	0,52	168,35 DH
Ascites puncture	323,75 DH	0,47	152,16 DH
injection of the drug	323,75 DH	0,05	16,19 DH

The cost of a chemotherapy procedure is equal to the cost per relative unit since the procedure has been assigned a value of 1.

## Discussion

Through a three-step process, it was possible to allocate indirect costs in order to identify the cost per department (oncology service) and per product (chemotherapy act, transfusion, ascites puncture, hospitalization day).

In the first step, we identified and allocated all indirect costs to the ONCO center (medical oncology department), which was the subject of the cost analysis, using the appropriate allocation bases.

In the second step, we allocated the various indirect costs identified in the first step to the units of the oncology department (HDJ unit and HOSP unit), using the allocation bases of the second step.

In the third step, we determined the indirect unit cost per product (hospital day, chemotherapy procedure, etc.). The cost of the day of hospitalization in the hospitalization unit is determined more or less easily because the method used is the volume-based costing method. It is equal to the total cost of the inpatient unit divided by the total number of inpatient days.

Indeed, the volume-based costing (VBC) method is widely used in the hospital context [6]. It uses volume units, mainly hospital days, to allocate costs to patients [7].

However, determining the indirect cost of medical procedures performed in the HDJ unit is relatively difficult because these procedures are not homogeneous. For this purpose, we used the relative value unit method to determine the unit cost of the different medical procedures performed in HDJ. This cost corresponds to the division of the total indirect cost of the HDJ unit by the number of work units produced.

The cost calculation method proposed in this study follows a top-down approach. Indeed, the top-down costing approach has been advocated for middle- and low-income countries in order to carry out costing studies while waiting for the implementation of a cost accounting system [8]. In this approach, costs are first distributed to services and then to stays using a distribution key, which leads to an average cost per patient [9].

In contrast, the bottom-up approach is considered more accurate, but complex compared to the top-down approach. It focuses on differences in patient care time, as it generates costs based on resource use, which can vary over time and between individuals. Activity-based costing is the most widely used method for calculating bottom-up costs [10].

The review of hospital costing systems used in several countries revealed the widespread use of traditional hospital costing methods. Traditional costing methods (volume-based allocation, RCC, RVU) have proven to be very successful in the hospital setting in several countries.

The example of the American model, which uses mainly traditional costing methods, is more in line with institutional requirements, mainly the Medicare prescribed cost report. Indeed, the regulatory principles of costing had an influence on the configuration of hospital costing systems. A contractual agreement (participation agreement) is required when a hospital wishes to become a participant in the Medicare program [11].

Two main methods have been advocated for determining the cost per product in the program. One is the volume-based allocation method and the other is the RCC method.

With the advent of the prospective payment system and the need to control high hospital expenditures, the RVU (relative value unit) approach has become a standard methodology for determining the cost of medical procedures for a number of institutions. Determining the costs of individual procedures is a time-consuming and complex task that requires the cooperation of many people at the hospital level. Thus, the associated cost can be very high. In this regard, state hospital agencies frequently calculate a standardized set of relative value units to which hospitals can refer [12].

Also, the French hospital costing system is prescribed by the supervisory authorities [13]. The French hospital cost accounting (CAH) is largely based on the traditional American model of cost calculation by DRG. It has undergone considerable changes in connection with the reforms undertaken in hospital financing [14].

Hospitals have shown increasing interest in hospital cost accounting since the introduction of two major reforms: organization into clusters and activity-based pricing (T2A). CAH makes it possible to isolate the expenses that can be allocated to the clusters, to identify internal situations of financial imbalance and to make decisions about the allocation of resources within the hospital or within a cluster [15]. T2A consists of paying hospitals according to their activity measured by homogeneous group of patients.

A corollary of the homogeneous section approach advocated by the CAH is the definition of two distinct types of analysis sections: definitive analysis sections and ancillary analysis sections. The cost of clinical services is allocated to each patient stay through the use of the volume-based allocation method. The relative cost index (ICR: equivalent to the RVU) is used to allocate the costs of medical-technical departments to each patient stay. The ICR is considered a relative index of resource mobilization, allowing procedures to be positioned in relation to each other.

Similarly, the choice of traditional costing methods instead of activity-based costing methods is explained by the following reasons:

Traditional costing methods (volume-based allocation, RCC, RVU) are widely used in the international hospital setting compared to activity-based methods (ABC and TDABC) [16].

The objective of activity-based methods is to obtain more relevant costs through a better allocation of indirect costs. The studies on the application of ABC or TDABC are based on a comparative vision with traditional costing methods in order to highlight the shortcomings and benefits obtained from the implementation of an activity-based approach. An example of this is the study by Yun et al. [5] who applied the TDABC method to determine the costs of selected services performed at the emergency department level. This study found differences in costs when using the three methods: RCC, RVU and TDABC.

Attempts to develop and sustain ABC have proven difficult. Indeed, the difficulty of applying ABC to an entire institution is linked to the cumbersome nature of its implementation and the cost of the necessary information. It has thus required significant investments in the resources of organizations, which has led to partial or incomplete applications [17].

The TDABC was presented by Kaplan and Anderson [18] as a modified version, simple and less complex than the ABC. Kaplan and Porter presented a seven-step approach to implementing TDABC in health care facilities as a solution to the cost crisis, and linked it to the VBHC program [19].

However, studies of TDABC implementation have aimed to provide more accurate cost estimates to assist in the move toward new funding models, such as bundled payment in the United States [20]. This mode involves assigning global reimbursement for a complete care pathway to all actors involved in the patient care process. A second main objective of the TDABC is to identify opportunities for cost optimization to reduce health care spending, which has increased exponentially in the United States, and to shift more financial risk to providers. However, the Moroccan context is very different from the US context. Health facility financing methods are still considered traditional. They are based primarily on an online budget allocation and fee-for-service payment [21].

From a practical standpoint, we found difficulties in allocating operating costs to activities. Medical oncology is characterized by the presence of many activities. Using multiple cost drivers will make the model more cumbersome and complex.

**Fig. 3:**
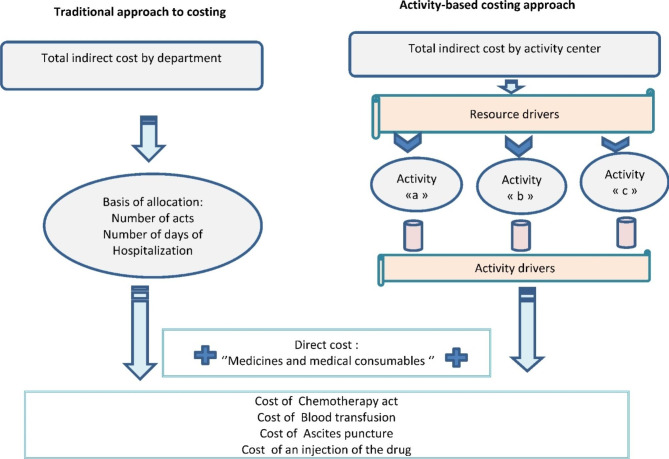
Application of the traditional and activity-based approaches

The Fig. 3 shows the application of the two costing approaches in the hospital context: traditional and activity-based:

The choice of costing approach is dictated primarily by the purpose of the study. Activity-based costing methods are mainly used if the objective is to obtain precise costs and if the means are available to carry out complex projects (adapted information system, important technical and human means…). However, in this study, we were more interested in the feasibility of a cost calculation method than in obtaining very precise results.

We have therefore chosen to use a top-down approach, recommended for the hospital context in low- and middle-income countries, to carry out cost studies.

This approach will make it possible to identify the costs of services considered to be priorities, pending the implementation of a system of classification and calculation of costs by diagnostic group [22]. We also used two costing methods that have proven successful in the hospital setting: the volume-based allocation method and the RVU method.

The approach proposed for the calculation of costs is considered simple to implement insofar as it is based on a top-down approach that consists of allocating indirect costs to the ONCO center (oncology department) and then to the HDJ and HOSP unit, as well as to the object of the cost (chemotherapy procedure, blood transfusion, etc.) using volume allocation bases.

Concerning the capacity of the approach to produce precise costs, we can admit that the methodology developed is made up of several stages in each of which is used a modality of allocation of indirect expenses.

The allocation of indirect costs in our study context is very delicate and is also marked by a significant degree of subjectivity.

Of course, the cost calculation process developed is a combination of several calculation and accounting methods. The determination of the cost per service, per unit, and then per medical act will be able to produce data that will help in the management of the oncology service. In other words, the methodology developed invites the actors to question their organization, their information system and their management mode.

## Conclusion

Hospital reforms are directed towards rationalization and better use of resources in health care services in order to reduce costs while maintaining or improving the quality of care provided to patients.

In Morocco, the literature review shows that there is little work on the calculation of the costs of hospital services.

In the absence of a hospital cost accounting system, we tried to propose a costing approach for the medical oncology department of the University Hospital of Fez. To do this, it was necessary to understand the organization and the course of the activities of the medical oncology service and to measure the resources mobilized to ensure the various care services.

One of the main results of the study is the possible application of a top-down approach to calculate the costs of medical acts performed in the medical oncology department. This approach is primarily based on traditional costing methods.

In this regard, two main costing methods were exploited: the volume-based allocation method and the RVU method.

The proposed top-down cost allocation approach has the advantage of being easy to implement, but on the other hand, it is not very accurate in producing results on the actual cost. The main cause of this inaccuracy is the way indirect costs are allocated to the cost object, using volumetric allocation bases, whether the cost is determined by department, unit or medical procedure. For example, the cost of a day of hospitalization is determined by dividing the total indirect cost of the inpatient unit by the number of days of hospitalization.

Another limitation of this study is its generalizability. Indeed, the cost data for the medical oncology center are for a teaching hospital where some expenses, such as those for personnel and other operating costs, may be high compared with expenses used in regional oncology centers or private clinics.

It would therefore be desirable to extend this cost study to other oncology centers in order to seek standardization in terms of methodology and to compare the costs determined. In addition, the determination of the average cost per medical procedure will serve the process of setting new national tariffs.

Thus, cost data on medical oncology services will be used to develop new national tariffs and to feed hospital management tools, including planning, budgeting and hospital dashboards.

In addition, hospital managers and researchers in low- and middle-income countries can use the proposed approach to implement cost studies of different health care services.

## Data Availability

Not applicable.
